# Polydrug Use among Students in a Public University in a Lower Middle-Income Country

**DOI:** 10.1155/2023/8085588

**Published:** 2023-08-01

**Authors:** Joan Jelagat, Nancy L. M. Budambula, Moses Ngari, Valentine Budambula

**Affiliations:** ^1^Department of Environment and Health Sciences, Technical University of Mombasa, Kenya; ^2^Department of Biological Sciences, University of Embu, Kenya; ^3^KEMRI-Wellcome Trust Research Programme, Kilifi, Kenya; ^4^Department of Public Health, Pwani University, Kenya

## Abstract

Recreational drug use among students in tertiary institutions remains a public health concern. Despite documentation of drug use in Kenyan universities, most of the studies are based on self-reported history which is prone to social desirability bias. It is in this context that we sought to establish lifetime and current drug use among university students. The study investigated self-reported and confirmed drug use. Using proportionate to size and snowball sampling methods, 380 respondents were enrolled from three university campuses. Actual drug use was confirmed qualitatively using a 6 panel plus alcohol saliva test kit. The study participants' median (IQR) age was 22 (20–23) years, and 262 (69%) were male; 328 (86%) were degree-level students, while 127 (33%) were in their fourth year and above. A total of 221 (58%) students reported a lifetime ever use of drugs, while 193 (51%) tested positive for at least one drug. Alcohol, tobacco products (cotinine), marijuana, and amphetamine or khat were the most preferred drugs. The usage was either solely, concurrently, or simultaneously. Having multiple sexual partners compared to students with no sexual partner (adjusted risk ratio (aRR) of 2.33 (95% CI 1.45, 3.76)) and residing in Mishomoroni and Kisauni (aRR 1.50 (95% CI 1.08, 2.09)) were associated with risk of testing positive for any drug. Having one (aRR of 1.54 (95% CI 1.05, 2.26)) and multiple sexual partners (aRR 2.03 (95% CI 1.27, 3.25) and residing in Mishomoroni and Kisauni (aRR 1.48 (95% CI 1.05, 2.08)) were associated with self-reported drug use. One out of two students was currently using drugs. Irrespective of the method used to record data, alcohol, tobacco products, marijuana, and amphetamine or khat were the most preferred drugs. The usage was solely, concurrently, or simultaneously. Future interventions should focus on continuing students, students' residences, and those who are sexually active.

## 1. Introduction

Substance use, abuse, and addiction remain a major global public health problem. It is estimated that in 2021, about 296 million people worldwide aged 15-64 years had used drugs at least once, with *Cannabis* sp. being the most widely used illicit drug [1]. The peak age for drug use is between 18 and 25 years old, with the youth in sub-Saharan Africa being the most affected [1, 2]. Kenya has not been spared by this crisis, as reported by the National Authority for the Campaign against Alcohol and Drug Abuse (NACADA). In this report, the prevalence of lifetime drug use was 57%, with alcohol being the most abused [3].

Most university students fall into the age bracket of 18 to 25 years. The behavioral vast majority of these students are transitioning into adulthood, living independently, and making behavioral health decisions without direct parental or adult oversight [4–8]. Throughout the USA, in 49 medical colleges, 91.3% and 26.2% of medical students reported to have used alcohol and marijuana, respectively, in the past year [8]. In the mid-Atlantic USA, marijuana was the most reported commonly used drug among college students, with the highest annual peak occurring in year three of the study [9].

Among Norwegian students, a study using the Alcohol Use Disorders Identification Test (AUDIT) reported that approximately 53.0% of the students had an AUDIT score of eight or above. This level is categorized as hazardous drinking [10]. Some of the risk factors for drug use among Norwegian students included being a single male without children, being a native Norwegian, being nonreligious, being extroverted, and having failed in examinations more than once [10, 11]. Among university students in India, alcohol, tobacco, and *Cannabis* were the most preferred drugs, with a vast majority using drugs to relieve psychological stress, to celebrate special occasions, to reduce fatigue, and out of curiosity [12]. In Kuwait, drug use was positively associated with poor academic performance, high family income, being an only child, and having divorced parents [13].

In well-resourced countries, drug use among university students varies depending on the type of drug and ranges from 30 to 90%. For example, a national survey of 2810 students in the UK in 2018 reported that 56% of respondents had used drugs, while 39% were current users [14]. In Ireland and the United Kingdom, alcohol use ranges between 30 and 70% [15, 16]. In the USA, *Cannabis* use is more prevalent than other drugs and ranges from 30 to 90% [9, 17, 18]. Predictors of drug use among university students include majoring in social sciences and business studies, being male, living away from home, having a low level of health awareness, perceived stress, being nonreligious, and having a friend who uses drugs [4, 10, 11, 16, 19].

Drug use among university students is highly prevalent in Africa. For example, in Lagos (southwest Nigeria), a cross-sectional study reported a 20% lifetime alcohol use. Friends and peer pressure were the most common influencers of substance use in this subpopulation [20]. Findings from a university in the Western Cape (SA) reported a prevalence rate of 62.7%, with alcohol (80.6%) and *Cannabis* (46%) being the frequently used substances. Substance use was associated with depression [21]. In Rwanda, the prevalence of having ever smoked shisha among university students was reported to be 26.1% and associated with alcohol use [22].

On the other hand, in Sudan, being male was predictive of drug use, with tobacco, *Cannabis*, and alcohol being the most consumed drugs [23]. A meta-analysis of 24 studies in Ethiopia showed that khat chewing was rampant among university students. Being male, having a family that practiced khat chewing, and having a friend's khat chewing habits were predictive of drug use [24]. In Uganda, 39% of participants from a public university reported alcohol use. Regular alcohol use was significantly associated with moderate and high-level social media use [25]. In Tanzania, a cross-sectional survey among young people sampled from secondary schools, tertiary institutions, and out-of-school youths from Kilimanjaro and Mwanza regions reported the highest percentage of alcohol use among college students. The current alcohol use was 45% in males and 26% in females among college students. Overall, being male, in a relationship, a nonmuslim, having multiple sexual partners, and having greater disposable income were associated with alcohol use [26].

In the Middle East and North Africa, lifetime self-reported drug use ranges between 6.5% and 48.5% [27, 28]. Alcohol, tobacco products, *Cannabis*, and khat are the most commonly used drugs [27, 29–31]. Predictors of drug use are being male, being an only child, having poor academic performance, having a high family income, having divorced parents, having a positive history of family conflict, encouragement by peers, having a positive history of child abuse, having a stay-at-home mother, living in an urban area, living away from home, and having a family history of substance use [13, 29, 30, 32].

Substance use prevalence rate among university students in Africa has been reported to be as high as 62.7% to 84.5% [21, 33, 34]. The most commonly consumed substances are alcohol, tobacco products, and *Cannabis* [21, 33–36]. Predictors of drug use in this subpopulation include being male, residing off-campus, having a roommate or friend who is already using drugs, being sexually active, having multiple sexual partnerships, infrequent attendance at religious fellowships, and depression [26, 33, 35, 37].

Kenyan universities have not been spared the drug abuse menace either. For example, a 68.9% lifetime prevalence of at least one substance was reported among students from four tertiary institutions in Eldoret, a town in western Kenya. Additionally, lifetime use of alcohol and tobacco was approximately 51.9% and 42.8%, respectively [38]. A different study that sampled one university campus and three colleges in Eldoret reported a lifetime prevalence of substance use of 41.5%. Additionally, the lifetime prevalence of alcohol and cigarette use was 36% and 8.3%, respectively. In this cohort, substance use was heightened by personality traits, specifically being neurotic and having a low score on agreeableness [39]. Conversely, a study carried out among first-year students at a university in Nairobi reported alcohol (22%), *Cannabis* (8%), and tobacco (7%) to be the most frequently used drugs. In this study, living on campus was a protective factor against drug use [40].

In Kenya, the percentage range of lifetime self-reported drug use among university students is between 25 and 68.9% [38, 40], with alcohol, tobacco, and *Cannabis* being the most preferred drugs [38, 39]. Common predictors of drug use are being male, scoring high neuroticism and low agreeableness personality traits, having a moderate stipend, being encouraged by peers, and living off-campus [38–40].

Despite extensive documentation of drug abuse among university students, most of the studies are based on self-reported history which is prone to social desirability bias. It is for this reason that this study sought to determine drug use patterns using saliva testing as a confirmatory method.

## 2. Materials and Methods

### 2.1. Setting, Design, and Study Participants

This cross-sectional study sought to document recreational drug use among university students in coastal Kenya between February 2017 and July 2017. Using proportionate to size and snowball sampling methods, respondents were enrolled from three campuses of the Technical University of Mombasa, namely, the Main Campus located in Mombasa County, the Kwale Campus (in Kwale County), and the Lamu Campus (Lamu County). The snowball method has been used elsewhere in similar studies [41, 42]. The inclusion criteria for participation in the study were being a bona fide student at the time of the study, having verbal consent, being both male and female students above the age of 18 but below the age of 30, being an undergraduate, and having been on campus for more than one semester.

### 2.2. Sample Size Determination

At the time of the study, 51% of the residents of Mombasa County reported a life prevalence of at least one substance [43]. Assuming a 51% proportion of university students using drugs and a level of significance of 5%, we required a sample of 384 students [44]. At the time of the study, the total population of students in the university was 16,258 and was distributed as follows: Main Campus (9,429 students), Kwale Campus (4,877 students), and Lamu Campus (1952 students). Based on proportion to size, we recruited 223 participants from the Main Campus, 115 from the Kwale Campus, and 46 from the Lamu Campus through a snowball sampling method. Out of the 384 participants, four declined to consent to saliva testing and were excluded from the final analysis.

### 2.3. Data Collection Tools

Social demographic characteristics and self-reported drug use history were documented using a self-developed participant-assisted questionnaire. We pretested the questions at the time of proposal development on students who were proceeding with field attachment. This cohort did not participate in the study as they were out of session. The questions were revised accordingly.

Actual drug use was determined qualitatively using the 6 panel plus alcohol SalivaConfirm™ Saliva Test kits (Confirm Biosciences) as per the manufacturer's instructions. These kits utilize monoclonal antibodies that detect high levels of selected drugs in human oral fluids. They utilize lateral flow chromatographic immunoassay for the qualitative detection of drugs. The six panel plus alcohol was used to detect amphetamine, cocaine, opiates, marijuana, benzodiazepines, cotinine, and alcohol. Amphetamine is structurally related to khat, while cotinine is a metabolite of nicotine. The kit consists of a mouth sponge swab and a test cup. Each participant was asked to insert the mouth sponge swab in the mouth either on the tongue or the cheek in order to soak the sponge swab with saliva. The soaked sponge swab was then inserted into the test cup and tightly closed. The results were interpreted after ten minutes. The appearance of one red line in the control section indicated positive results, whereas two lines, one at the control and the other at the test, translated as negative results. When no line appeared at the control or the testing section, it meant invalid results, and the participant would be requested to retake the test.

### 2.4. Ethical Considerations

The study was ethically reviewed and approved by the Pwani University Ethical Review Committee (ERC/Msc/034/2016). Informed verbal consent was obtained from each participant. Privacy and confidentiality were observed as the interview as well as testing took place in a private room. Only one participant was allowed into the room at a time. No name was recorded on the participant-assisted questionnaire or used anywhere in the study. Hard copies were stored in a lockable cabinet whose access is restricted to authorized personnel. The soft copy of the study has restricted access by the use of a password.

### 2.5. Statistical Methods

All categorical variables were reported as proportions with their respective percentages, while age was summarized using the mean and standard deviation. To examine the demographic features associated with confirmed positive test results for any drug, we used log-binomial regression analysis and reported transformed regression coefficient into risk ratios (RR) and their respective 95% confidence intervals. The dependent variable was the binary drug test results from saliva analysis (either positive or negative).

In the univariate models, we tested each demographic variable separately. In the multivariable regression analysis, we applied a backward stepwise approach to retain independent variables with a *P* < 0.1. Log-binomial regression analysis was preferred because logistic regression overestimates the measure of effects for a common outcome with a prevalence of more than 10% [45]. As a sensitivity analysis, we also a performed regression analysis of demographic factors associated with self-reported drug use to assess if they would be similar to those of saliva test-confirmed drug use. We assessed the multivariable regression model's performance using the area under receiver operating characteristics curve (AUC).

Statistical significance was assumed at *α* < 0.05. All statistical analyses were performed using STATA version 15.1 (StataCorp, College Station, TX, USA).

## 3. Results

### 3.1. Sociodemographic Characteristics of Participants

We recruited 380 participants whose median (IQR) age was 22 (20–23) years, and 118 (31%) were female. The majority (86%) of the study participants were enrolled in undergraduate degree programmes. Study participants were distributed as follows: 18%, 19%, 29%, and 33% in the first, second, third, and fourth years of study, respectively. In this study, participants who were first born by birth order were 28%, and 116 (31%) of participants were from the school of business. In addition, 95% reported never having been married, and 45% received a pocket money < USD 50 per month. One hundred and eight (28%) reported having pocket money that was above USD 50 but below USD 100 per month. Two hundred and sixty-two (61%) and 52 (14%) had one and multiple sexual partners, respectively. Two hundred (72%) had used a condom during their last sexual intercourse, as shown in Table 1.

### 3.2. Drug Use Patterns Based on Self-Reported History

**Figure 1:**
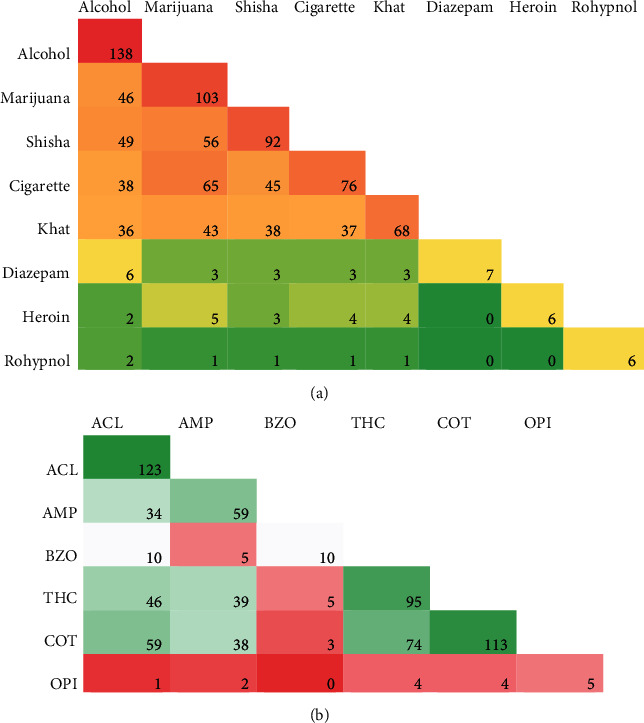
Combinations of self-reported used drugs (a), drugs detected by saliva test (b), and polydrug use among students attending a public university in Mombasa, Kenya, in 2017. The figures are heat map showing the intensity/numbers of drug combinations. ACL: alcohol; AMP: amphetamine; BZO, benzodiazepines; THC: tetrahydrocannabinol; COT: cotinine; OPI: opiates.

Of the 380 students, 221 (58%, 95% CI 53 to 63%) reported to have ever used drugs in their lifetime. One hundred and thirty-eight (62%) participants reported alcohol use, 103 (47%) had used marijuana, 92 (42%) had consumed shisha, 76 (34%) had ever smoked a cigarette, and 68 (31%) had ever chewed khat. Polydrug use among these participants was best described using a 5-class solution as used by Chen WL and Chen JH (19). The following drug combinations were the most popular: alcohol-marijuana-shisha-cigarette-khat; alcohol-marijuana-shisha-cigarette; alcohol-marijuana-shisha; alcohol-marijuana; and lastly, alcohol-marijuana-shisha-cigarette-khat-diazepam. These findings are summarized in Table 2 and Figure 1(a), respectively.

### 3.3. Confirmed Drug Use Patterns Based on Saliva Tests

Qualitative saliva analysis indicated that 193 (51%, 95% CI 46 to 56%) were positive for at least one drug. The results were as follows: alcohol (64%), cotinine (59%), tetrahydrocannabinol (49%), amphetamine (31%), benzodiazepines (5.2%), and opiates (2.6%). Out of the 193 participants who tested positive, 35.2% were monodrug users, 31.6% were positive for two drugs, 22.3% used three drugs, 9.8% four drugs, and one percent (1%) was positive for five drugs. The common drug combinations were as follows: alcohol-amphetamine-benzodiazepines-THC-cotinine; alcohol-amphetamine-benzodiazepines-THC; alcohol-amphetamine-benzodiazepines; alcohol-amphetamine; and alcohol-amphetamine-THC-cotinine-opiates (Table 2 and Figure 1(b)).

### 3.4. Sociodemographic Factors Predicting Drug Use

Based on the confirmed drug, univariate regression analysis predicted that age in years, birth order, year of study, students' residence, and number of sexual partners were associated with the risk of testing positive for any drug. Being a third-born predicted drug use with a crude risk ratio (CRR) of 1.36 (95% CI 1.03, 1.79). Students aged 22 to 24 years (CRR 1.49 (95% CI 1.21, 1.84) compared to those aged < 22 years were associated with a higher risk of testing positive for any drug. In comparison to participants in year one of the study, participants in years two, three, and four of the study were associated with a higher risk of testing positive for any drug, with a CRR of 1.60 (95% CI 1.08, 2.37), 1.76 (95% CI 1.22, 2.54), and 1.49 (95% CI 1.02, 2.17), respectively. Compared to residence in the university hostel and its environs, residence in Mishomoroni and Kisauni was associated with a risk of testing positive for any drug: CRR 1.94 (95% CI 1.37, 2.73). Compared to students with no sexual partner, having one or multiple sexual partners was associated with an adjusted risk ratio of 1.67 (95% CI 1.12, 2.49) and 2.58 (95% CI 1.62, 4.13), respectively. In the multivariable regression model, the effect of age in years was attenuated. However, having two or more sexual partners was associated with the risk of testing positive for any drug with an adjusted risk ratio (aRR) of 2.33 (95% CI 1.45, 3.76). Compared to residence in the university hostel and its environs, residence in Mishomoroni and Kisauni was associated with the risk of testing positive for any drug: aRR 1.75 (95% CI 1.23, 2.48). The multivariable regression model AUC was 0.77 (95% CI 0.72, 0.81), as summarized in Table 3.

Based on self-reported history, univariate regression analysis indicated that age in years, the year of study, amount of pocket money, student residence, and the number of sexual partners were associated with a history of lifetime drug use. Students aged 22 to 24 years (CRR 1.35 (95% CI 1.13, 1.61) compared to those aged < 22 years were associated with a higher risk of self-reported history of drug use. Compared to students in year one of the study, students in years two, three, and four of the study were associated with a higher risk of a history of lifetime drug use with crude risk ratios of 1.48 (95% CI 1.05, 2.07), 1.58 (95% CI 1.15, 2.16), and 1.45 (95% CI 1.06, 2.00), respectively. Compared to residence in the university hostel and the university environs, residence in Mishomoroni and Kisauni was associated with the risk of self-reported history of drug use: CRR 1.70 (95% CI 1.23, 2.34). Having pocket money between USD 50 and USD 100 was associated with a crude risk ratio of 1.42 (95% CI 1.05, 1.91) compared to pocket money that was less than USD 50 per month. However, in the multivariable regression model, the effect of age and amount of pocket money was attenuated. Compared to students with no sexual partner, having one (aRR 1.54 (95% CI 1.05, 2.26) or multiple (aRR 2.03 (95% CI 1.27, 3.25) sexual partners were associated with a higher risk of self-reported history of drug use. While compared to residence within the university hostel and its environs, residing in Mishomoroni and Kisauni was associated with the risk of self-reported drug use: aRR 1.50 (95% CI 1.08, 2.09). The multivariable regression model AUC was 0.79 (95% CI 0.74, 0.83), as summarized in Table 4.

## 4. Discussion

The overall prevalence of lifetime ever use and confirmed current use of at least one drug was 58% and 51%, respectively. Based on self-reported history, participants were likely to use alcohol, marijuana, tobacco products, and khat. Confirmatory tests agreed with reported drug use, as most participants tested positive for alcohol, cotinine, tetrahydrocannabinol, and amphetamine. Predictors of drug use were being a third-born child, having a year of study, receiving pocket money ranging between USD 50 and USD 100, and being sexually active. This was applicable for both self-reported history and confirmed drug use.

Alcohol was the most consumed drug in this study. The high prevalence of alcohol usage concurs with the national report that alcohol is the most used drug in Kenya [3]. Similarly, alcohol has been shown to be the most preferred drug by university students in Kenya [38, 40], while in Ethiopia [34] and Uganda [35], almost half of the students consume alcohol. Outside Africa, the prevalence rate of alcohol consumption among university students ranged from 19.9% to 33% in Iran, Ireland, and the United Kingdom [15, 16, 46].

The rampant use of alcohol by university students can be attributed to the fact that alcohol is considered a gateway drug [47]. Additionally, the college environment could also be playing a role in alcohol consumption. Some of the risky environmental factors include living in a hostel with a higher number of roommates, partying, and environmental cues [48, 49]. The high rate of alcohol use could be due to its legality [50] which makes alcohol easily available, accessible, acceptable, and affordable.

In this study, tobacco products were the second most popular drug used, and students were likely to smoke cigarettes or shisha. This was supported by the participants testing positive for cotinine. In Kenya, a lifetime prevalence rate of tobacco use of 42.8% has been reported among students in tertiary institutions in Eldoret [38], while in Rwanda, a prevalence of 26.1% was reported [22]. Elsewhere, in Sudan and Egypt, tobacco use was most prevalent among university students at 13.7% and 8.9%, respectively [23, 27], while smoked and nonsmoked tobacco were some of the most preferred drugs by university students in India [51].

The present study reports marijuana use by university students. These findings concur with results documented in South Africa as well as Egypt, where 30.9% and 24.3% of students reported lifetime use of *Cannabis* [27, 36]. In the mid-Atlantic (USA), marijuana was the most commonly used drug among college students in every year of the study [9]. Likewise, across 49 medical colleges in the USA, about 26.2% of the medical students had used marijuana in the past one year [8]. The possession, sale, growing, and distribution of marijuana in Kenya are illegal [52]. Despite this, marijuana is unlawfully grown in Kenya, making it relatively easily available and accessible. Of great concern, especially in learning institutions, are the effects induced by delta-9-tetrahydrocannabinol (THC) on verbal, thinking, and working memory [53].

We report the use of khat in the present study. A previous study reported 14.6% lifetime use in Ethiopia [54]. Findings indicating a high prevalence of khat use are similar to those of a systematic review that reported khat as one of the most consumed substances among university students in Ethiopia [55]. Further, a meta-analysis indicated that the pooled prevalence of current khat use among university students was highest in Saudi Arabia (18.9%), followed by Ethiopia at 13.6%, and 13% in Yemen [31]. Testing positive for amphetamine could be due to chewing khat which is a common practice in Kenya and more so in Mombasa. Khat seems to be an emerging gateway drug.

In the present study, a significant proportion of university students who tested positive were polydrug users. Irrespective of the method used to record data, alcohol, tobacco products, marijuana, and amphetamine or khat were the most preferred combinations. Polydrug use has been previously reported in a Kenyan university, with the main combination being *Cannabis*, tobacco, and alcohol [40]. In Ethiopia, khat use was strongly and positively associated with alcohol consumption [56]. Concurrent use of *Cannabis* and tobacco has been previously reported among university students in Spain and Brazil [57, 58].

Polydrug use could be for purposes of enhancing the potency of a drug, reducing its side effects, or alleviating withdrawal symptoms. For example, drinking alcohol immediately after smoking *Cannabis* intensifies the effects of the latter. This is so because alcohol increases the absorption of THC, thus enhancing plasma THC levels [59]. Polydrug use among university students can also be attributed to the college environment which not only facilitates drug acquisition but also contains environmental cues that trigger substance cravings [48].

In this subpopulation, being a third- or fourth-born child increased the risk of drug use, while being a second-born was protective. These results corroborate the findings of a study carried out in the Netherlands among adolescents, whereby acute alcohol intoxication was more likely to occur in participants who had an older sibling. Thus, being the only child or the first born child was considered a moderating factor for acute alcohol intoxication [60]. Similarly, in Sweden, after-born siblings had a higher likelihood of getting hospitalized for both alcohol and narcotics use [61]. This could be because the latter-born children are likely to be exposed to drugs at younger ages through older siblings.

The year of the study predicted drug use, as participants in the second, third and fourth years and above had an elevated risk of substance consumption. Similar findings were reported in Ethiopia, where being in the third year of study compared to the first year increased the odds of substance use [62]. In Sudan, being in the third year carries a higher risk of drug use than being in the fifth year of study [23]. Findings on the association between the third year of study and drug use are comparable to an eight-year longitudinal analysis in the mid-Atlantic that reported the highest annual peak of marijuana use in year three of the study [9].

This could be attributed to factors like academic-related stress [63], poor social skills, thus increased vulnerability to peer pressure to use drugs [64], limited physical activities [65], and poor quality dating relationships [66]. All the aforementioned factors combined increase vulnerability to stress and depression which in turn heighten drug use as a form of self-medication. Moreover, based on general observations, most universities tend to pay more attention to students in their first year of training than students in subsequent years. This is evidenced by the matriculation ceremony and life skill-related courses that are mandatory in the first year. Additionally, most parents tend to lose touch with their children as they progress into their senior years because they perceive the latter to have matured. Less parental closeness has been reported to be a predictor of substance use [67].

Living within the university hostels or around the university was a moderating factor in this study. On the other hand, students who lived away from the university around town were at the highest risk of drug use. This concurs with the findings of a study at the University of Nairobi in Kenya, whereby students living off campus were more likely to use drugs as compared to their on-campus counterparts [40]. Similar results were reported among university students in the Northeast of the United States, where living off campus posed the highest risk of alcohol use [4]. This could be attributed to the fact that students living off-campus are more likely to reside on the low-income side of town. Poor neighbourhoods act as a haven for drug peddlers and illicit liquor breweries, making drugs easily accessible.

There was a higher risk of drug use among students with a moderate stipend (USD 50 and USD 100) than among those with extremely low income (<USD 50) or very high income (>USD 100). Extreme poverty and superfluity seem to be a moderating factor in this study. These findings differ from the results of a study across 23 public and private USA institutions that reported undergraduates with high socioeconomic status were more likely than peers to use marijuana as well as practice polydrug use [68]. Similarly, among university students in Ethiopia, having a higher monthly income predicted drug use [69]. Among high school students in North Carolina, having a weekly disposable income over USD 50 was a correlate of current hookah use [70]. In Kuwait, students with high family income were more likely to use shisha [71]. This could be attributed to the fact that drug use has a cost implication.

In the present study, drug use was associated with having one or more sexual partners. These findings correspond to the results of a study carried out at Mbarara University in Uganda, where students who reported alcohol use were more likely to have multiple sexual partners [35]. A positive association between higher use of khat as well as tobacco products and multiple sexual partnerships has also been reported in Ethiopia [71]. Outside Africa, university students from nine countries belonging to the Association of Southeast Asian Nations (ASEAN) with multiple partners were significantly more likely to be current tobacco smokers and binge drinkers [72]. Additionally, a systematic review reported an amplified association between alcohol and risky sexual behaviors among university students [73]. This association could be linked to the fact that drugs like alcohol reduce inhibition towards sex, while *Cannabis* is a vasodilator, thus enhancing sexual libido [74, 75].

This being a cross-sectional study, it is difficult to determine whether the outcome followed exposure in time or whether exposure resulted from the outcome. The strength of the study is anchored in confirmed drug use. This study was able to detect drugs which are currently being consumed using saliva analysis. To the best of our knowledge, this is the first study in Kenya to document validated drug use among university students. Although saliva analysis is not prone to social desirability bias, it only detects drugs used in the past few days, hence the limitation.

## 5. Conclusions

The lifetime ever use of drugs was 58% based on self-reported history and 51% when determined by saliva tests among university students at three university campuses. Being a latter-born, in the third year of study with moderate income, residing outside the university hostel or its environs, and having multiple sex partners were strong predictors for drug use. Irrespective of the method used to record data, alcohol, tobacco products, marijuana, and amphetamine or khat were the most preferred drugs. The use of these drugs was solely, concurrently, or simultaneously.

An integrated approach to preventing substance use among this young population is warranted and crucial. Future interventions should focus on students who have been on campus for a longer duration and those who are sexually active. Policymakers and university management should prioritize the expansion of on-campus accommodation. The findings of this study may be used as baseline data for future studies. We recommend comparative studies be done using self-reported data, urine, blood, and hair analyses.

## Figures and Tables

**Table 1:** Selected characteristics of students attending a public university in Mombasa, Kenya, in 2017.

Sociodemographic characteristics	Total (*N* = 380)
Age in years	
<22	164 (43)
22 to 24	174 (46)
>24	42 (11)
Gender, *N* (%)	
Male	262 (69)
Female	118 (31)
Education level, *N* (%)	
Degree student	328 (86)
Diploma student	52 (14)
Birth order, *N* (%)	
First born	107 (28)
2nd born	77 (20)
3rd born	74 (20)
4th born and above	122 (32)
School enrolled in, *N* (%)	
School of business	116 (31)
School of applied and health sciences	100 (26)
School of engineering	89 (23)
School of humanities & social sciences	49 (13)
Institute of computing and informatics	26 (6.8)
Year of study	
First year	68 (18)
Second year	74 (20)
Third year	111 (29)
Fourth year and above	127 (33)
Residence or accommodation type, *N* (%)	
University hostel	34 (9.0)
University environs	110 (29)
Mishomoroni and Kisauni	84 (22)
Within town (Makupa, Sparki, & Majengo)	116 (31)
Bamburi and Bombolulu	36 (9.5)
Marital status, *N* (%)	
Never married	361 (95)
Married/cohabiting	19 (5.0)
Income/pocket money per month (USD), *N* (%)	
<50	170 (45)
Between 50 and 100	108 (28)
>100	102 (27)
Current number of sexual partners, *N* (%)	
None	96 (25)
One	232 (61)
Two and above	52 (14)
Used condom during last sexual intercourse-yes (%)	200 (72)

Results are *N* (%) unless specified.

**Table 2:** Self-reported drug use and saliva drug tests in a sample of students in a public university in Mombasa, Kenya, in 2017.

Reported drugs, *N* (%)	*N* = 221
Alcohol	138 (62)
Marijuana	103 (47)
Shisha	92 (42)
Cigarette	76 (34)
Khat	68 (31)
Diazepam	7 (3.2)
Heroin	6 (2.7)
Rohypnol	6 (2.7)

Drugs tested positive, *N* (%)	*N* = 193
Alcohol (ACL)	123 (64)
Cotinine (COT)	113 (59)
Tetrahydrocannabinol (THC)	95 (49)
Amphetamine (AMP)	59 (31)
Benzodiazepine (BZO)	10 (5.2)
Opiates (OPI)	5 (2.6)

**Table 3:** Univariate and multivariable analyses of features associated with university students in Mombasa, Kenya, testing positive for any drug in 2017.

	Univariate analysis		Multivariable analysis	
	Crude RR (95% CI)	*P* value	Adjusted RR (95% CI)	*P* value
Sociodemographic characteristics				
Age in months				
<22	Reference		Reference	
22 to 24	1.49 (1.21, 1.84)	<0.001	1.30 (0.96, 1.77)	0.09
>24	0.67 (0.40, 1.11)	0.12	0.64 (0.35, 1.18)	0.15
Gender				
Male	Reference		¶	
Female	1.08 (0.87, 1.32)	0.49	¶	
Education level				
Degree student	Reference		¶	
Diploma student	0.94 (0.69, 1.27)	0.68	¶	
Birth order				
First born	Reference		¶	
2nd born	1.04 (0.76, 1.43)	0.80	¶	
3rd born	1.36 (1.03, 1.79)	0.03	¶	
4th born and above	1.17 (0.89, 1.53)	0.26	¶	
Faculty enrolled				
Business studies	Reference		¶	
Applied sciences	0.93 (0.69, 1.24)	0.62	¶	
Engineering	1.26 (0.97, 1.62)	0.08	¶	
Humanities & social sciences	1.12 (0.81, 1.55)	0.50	¶	
Computing & informatics	1.22 (0.83, 1.78)	0.31	¶	
Year of study				
First year	Reference		¶	
Second year	1.60 (1.08, 2.37)	0.02	¶	
Third year	1.76 (1.22, 2.54)	0.003	¶	
Fourth year	1.49 (1.02, 2.17)	0.04	¶	
Residence or accommodation type				
University hostel and its environs	Reference		Reference	
Mishomoroni and Kisauni	1.94 (1.37, 2.73)	<0.001	1.75 (1.23, 2.48)	0.002
Within town^∗^	0.99 (0.67, 1.44)	0.94	1.04 (0.71, 1.52)	0.84
Bamburi and Bombolulu	1.69 (0.93, 3.09)	0.09	1.80 (0.98, 3.29)	0.06
Marital status				
Never married	Reference		¶	
Married/cohabiting	0.82 (0.48, 1.41)	0.47	¶	
Income/pocket money/month, USD				
<50	Reference		¶	
Between 50 and 100	1.36 (0.99, 1.87)	0.06	¶	
>100	0.86 (0.60, 1.25)	0.44	¶	
Current number of sexual partners				
None	Reference		Reference	
One	1.67 (1.12, 2.49)	0.01	1.47 (0.98, 2.20)	0.06
Two and above	2.58 (1.62, 4.13)	<0.001	2.33 (1.45, 3.76)	<0.001

^∗^Makupa, Sparki, and Majengo. RR: relative risk; AUC: area under receiver operating characteristics; CI: confidence interval. The RR and *P* values are from a log-binomial regression model. The multivariate model AUC is 0.77 (95% CI 0.72, 0.81). 1 USD = Kshs 102; ¶: independent variables not selected for inclusion in multivariable.

**Table 4:** Univariate and multivariable analyses of features associated with self-reported drug use history of students in a public university in Mombasa, Kenya, in 2017.

Sociodemographic characteristics	Univariate analysis		Multivariable analysis	
	Crude RR (95% CI)	*P* value	Adjusted RR (95% CI)	*P* value
Age in years				
<22	Reference		Reference	
22 to 24	1.35 (1.13, 1.61)	0.001	1.21 (0.91, 1.61)	0.18
>24	0.64 (0.41, 1.01)	0.06	0.62 (0.35, 1.09)	0.09
Gender				
Male	Reference		¶	
Female	1.03 (0.86, 1.23)	0.76	¶	
Education level				
Degree student	Reference		¶	
Diploma student	0.99 (0.77, 1.27)	0.94	¶	
Birth order				
First born	Reference		¶	
2nd born	0.98 (0.75, 1.29)	0.89	¶	
3rd born	1.25 (0.99, 1.58)	0.07	¶	
4th born and above	1.09 (0.87, 1.37)	0.47	¶	
School enrolled				
School of business	Reference		¶	
School of applied & health sciences	0.94 (0.73, 1.21)	0.64	¶	
School engineering	1.22 (0.98, 1.52)	0.07	¶	
School humanities & social sciences	1.04 (0.77, 1.39)	0.81	¶	
Institute of computing & informatics	1.19 (0.86, 1.64)	0.31	¶	
Year of study				
First year	Reference		¶	
Second year	1.48 (1.05, 2.07)	0.02	¶	
Third year	1.58 (1.15, 2.16)	0.005	¶	
Fourth year and above	1.45 (1.06, 2.00)	0.02	¶	
Residence or accommodation type				
University hostel and its environs	Reference		Reference	
Mishomoroni and Kisauni	1.70 (1.23, 2.34)	0.001	1.50 (1.08, 2.09)	0.02
Within town^∗^	0.97 (0.69, 1.37)	0.88	1.07 (0.75, 1.52)	0.70
Bamburi and Bombolulu	1.39 (0.77, 2.50)	0.28	1.47 (0.81, 2.67)	0.20
Marital status				
Never married	Reference		¶	
Married/cohabiting	0.99 (0.67, 1.47)	0.98	¶	
Income/pocket money/month, USD USUSD (USD) (Kshs)				
<50	Reference		Reference	
Between 50 and 100	1.42 (1.05, 1.91)	0.02	1.28 (0.94, 1.73)	0.12
>100	0.88 (0.62, 1.25)	0.47	0.89 (0.62, 1.27)	0.51
Current number of sexual partners				
None	Reference		Reference	
One	1.73 (1.19, 2.51)	0.004	1.54 (1.05, 2.26)	0.03
Two and above	2.44 (1.57, 3.81)	<0.001	2.16 (1.37, 3.41)	0.001

^∗^Resident in Makupa, Sparki, and Majengo. RR: relative risk; AUC: area under receiver operating characteristics; CI: confidence interval. The RR and *P* values are from a log-binomial regression model. The multivariate model AUC is 0.79 (95% CI 0.74, 0.83); ¶: independent variables not selected for inclusion in multivariable.

## Data Availability

The data sets analyzed during the current study are available from the corresponding author on reasonable request.

## Abbreviations

ASEAN: Association of Southeast Asian Nations
AUDIT: Alcohol Use Disorder Identification Test
NACADA: National Authority for Campaign against Alcohol and Drug Abuse
THC: Tetrahydrocannabinol- the active chemical in *Cannabis* sp.
USA: United States of America
USD: United States dollar.
